# Effectiveness of Simulation‐based Training on Emergency Response Knowledge Among Inter‐Professional Staff Involved in Gastrointestinal Endoscopic Practice

**DOI:** 10.1002/deo2.70372

**Published:** 2026-07-01

**Authors:** Mitsuru Otsuka, Tsunetaka Kato, Takuto Hikichi, Jun Nakamura, Takumi Yanagita, Eisuke Kameoka, Daiki Nemoto, Rei Suzuki, Mitsuru Sugimoto, Hiroyuki Asama, Hiroshi Shimizu, Kento Osawa, Rei Ohira, Keisuke Kudo, Masao Kobayakawa, Hiromasa Ohira

**Affiliations:** ^1^ Department of Gastroenterology Fukushima Medical University School of Medicine Fukushima Japan; ^2^ Department of Endoscopy Fukushima Medical University Hospital Fukushima Japan; ^3^ Medical Research Center Fukushima Medical University Fukushima Japan

**Keywords:** emergency treatment, gastrointestinal endoscopy, interprofessional relations, simulation training, simulation‐based training

## Abstract

**Objective:**

To evaluate the effectiveness of simulation‐based training in improving emergency response capabilities during endoscopic practice.

**Methods:**

Physicians, nurses, laboratory technologists, and clinical engineers working in the endoscopy department were enrolled. The simulation‐based training program employed simulation models and was conducted under the guidance of a certified emergency nurse using predefined scenarios. Knowledge tests were administered before, immediately after, and 1 month after the training. Test scores were compared across time points and among professional groups.

**Results:**

Twenty‐two participants (14 physicians, seven nurses, and one laboratory technologist) were included. The accuracy rates improved across all items immediately after the training compared with the pre‐training outcomes: correct chest compression (*p* < 0.01), drugs for cardiac arrest (*p* < 0.01), drugs for anaphylaxis (*p* = 0.13), indications for defibrillation (*p* < 0.01), automated external defibrillator (AED) location (*p* = 0.01), and Code Blue contact number (*p* < 0.01). However, at 1 month after the training, most items returned to pre‐training levels, except for the indications for defibrillation and Code Blue contact number. In the subgroup analysis by profession, when the accuracy rates immediately before and immediately after the training were compared, physicians showed significant improvements in AED location (*p* = 0.03) and Code Blue contact number (*p* < 0.01), whereas nurses showed a significant improvement in drugs for cardiac arrest (*p* = 0.02).

**Conclusions:**

Simulation‐based training is effective in improving emergency response knowledge in gastrointestinal endoscopy. However, repeated or continuous training may be necessary to sustain these gains.

**Trial Registration**: N/A

## 1 Introduction

Gastrointestinal endoscopy is generally considered safe; however, adverse events, including bleeding, perforation, infection, allergic reactions, and cardiopulmonary complications, can occur. Among these, cardiopulmonary complications are reported in 0.02%–0.54% of cases and may lead to sudden clinical deterioration in patients [1, 2]. Recently, the demand for sedated endoscopy has increased, and sedation is now routinely used worldwide [3]. However, sedatives and analgesics may suppress the sympathetic nervous system activity, resulting in hypotension and respiratory depression, which can precipitate severe cardiopulmonary complications, including fatal outcomes [4, 5, 6]. The sedation guidelines of the American Society for Gastrointestinal Endoscopy (ASGE) [7], Japan Gastroenterological Endoscopy Society (JGES), and Japanese Society of Anesthesiologists (JSA) [8] recommend pre‐procedural assessment, including evaluation of general health status and airway anatomy, and appropriate monitoring during sedation to prevent the occurrence of adverse events. However, despite these preventive strategies, serious adverse events cannot be completely avoided [9]. Therefore, the Japanese sedation guidelines emphasize the importance of sedation‐related training, which includes Basic Life Support (BLS) and Advanced Cardiovascular Life Support (ACLS), to ensure appropriate responses to sudden clinical deterioration during sedation [8].

In several medical fields, including emergency medicine, pediatrics, and obstetrics/gynecology, simulation‐based training reportedly enhances self‐efficacy and knowledge during patient emergencies, including cardiac arrest [10, 11, 12]. Contrarily, simulation‐based training in gastrointestinal endoscopic practice has mainly focused on technical skills, including endoscope insertion and therapeutic procedures, and only a few studies have addressed the non‐technical skills in this setting [13, 14, 15, 16]. Only two studies have examined simulation‐based training for critical incident response in gastrointestinal endoscopic practice, with one evaluating self‐efficacy among physicians and nurses [17] and the other assessing knowledge levels using a written test among nurses and procedural technicians [18]. To the best of our knowledge, the present study is the first to objectively evaluate knowledge acquisition and retention using repeated knowledge assessments in a multidisciplinary simulation‐based training program, which also involves physicians, in gastrointestinal endoscopic practice.

Therefore, the present study aimed to evaluate the changes in the knowledge test scores before and after multidisciplinary simulation‐based training involving physicians and other professionals working in the endoscopy department and to assess the effectiveness of emergency response simulations in gastrointestinal endoscopic practice.

## 2 Methods

### 2.1 Study Design and Participants

The present study was conducted from January to February 2025 at Fukushima Medical University Hospital and included physicians, nurses, laboratory technologists, and clinical engineers involved in gastrointestinal endoscopic practice.

Participation in the simulation‐based training was voluntary. Among approximately 42 eligible healthcare professionals, 20 did not participate primarily because of scheduling conflicts, clinical duties, and limited staff availability during the training sessions.

Given that the present investigation did not constitute medical research involving human subjects according to the institutional regulations of Fukushima Medical University and did not involve patient data or medical interventions, prior approval from the Ethics Committee of Fukushima Medical University was not required. Participation was voluntary, and only anonymized knowledge test results were analyzed. No personal or sensitive information was collected. Participants were verbally informed that their knowledge test results would be used in the present study and subsequently provided verbal consent.

### 2.2 Questionnaire and Knowledge Test Within a Simulation‐based Training

**FIGURE 1:**
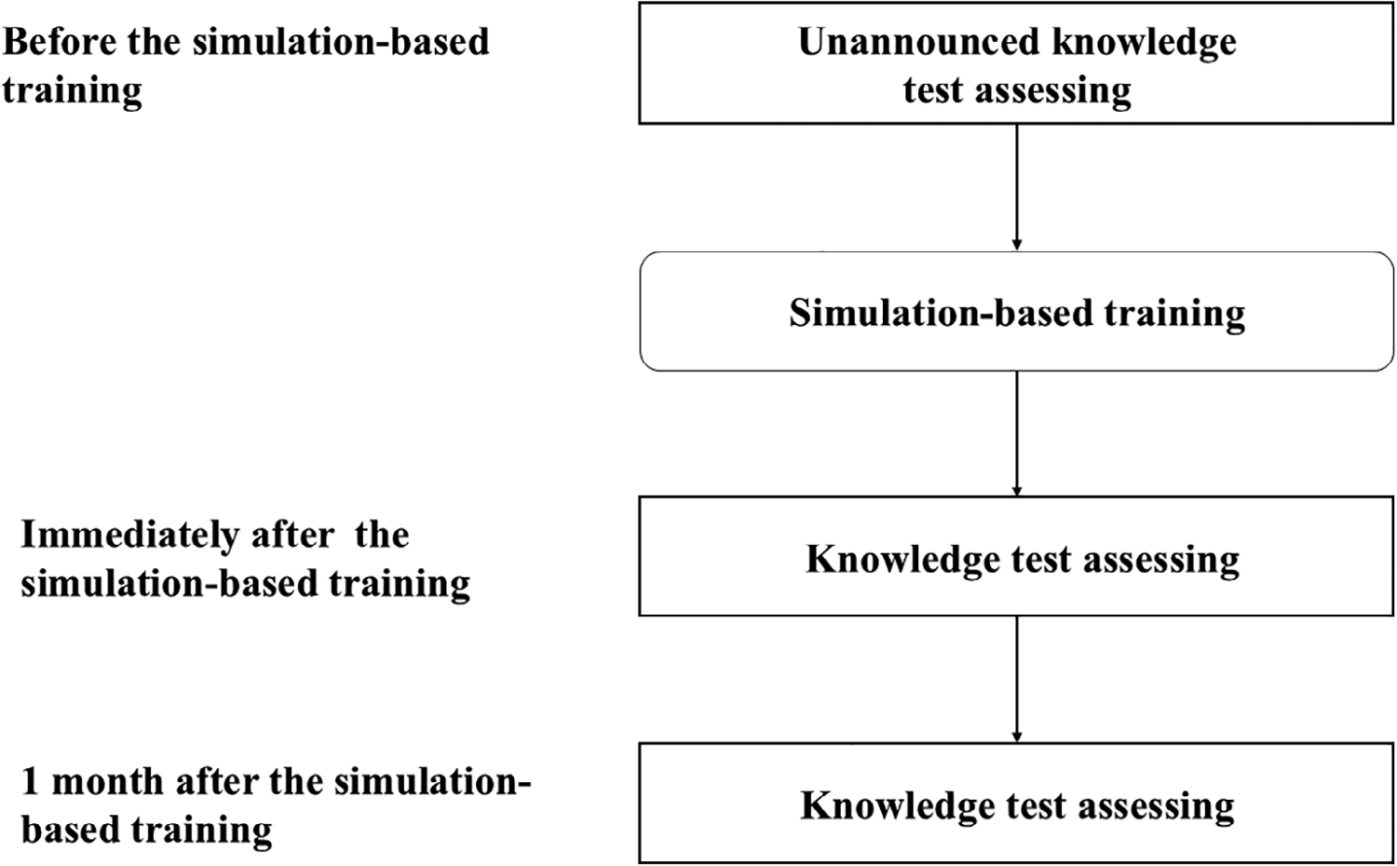
Flowchart of the present study.

The study flow is shown in Figure 1. Approximately 30 min before starting the simulation‐based training, a questionnaire and knowledge test (Table S1) were administered to the participants without prior notice. To prevent consultation among participants, all participants completed the questionnaire and knowledge test individually, and their answer sheets were subsequently collected. The knowledge test included questions aligned with the content of the simulation‐based training. Immediately after the 1‐h simulation‐based training session, within 30 min of its conclusion, the same knowledge test was individually administered to all participants on site, and the completed answer sheets were collected. At 1 month after the simulation‐based training, the same knowledge test was again individually administered to all participants without prior notice, and the completed answer sheets were collected.

### 2.3 Simulation‐based Training Contents and Procedures

**FIGURE 2:**
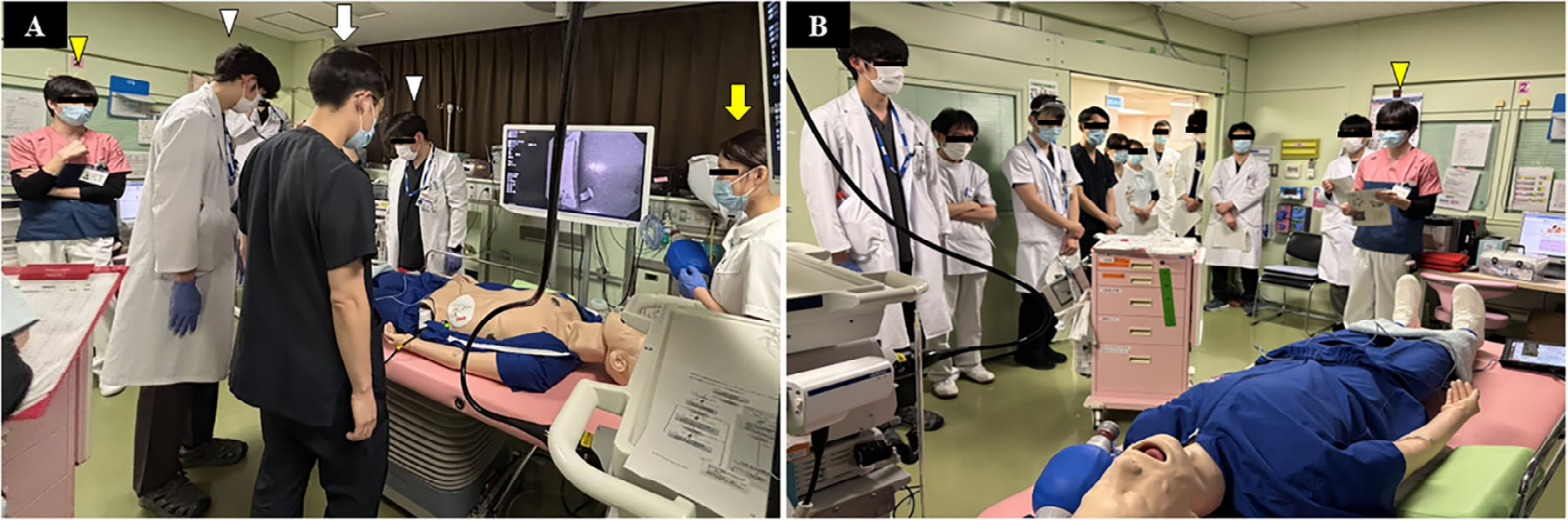
Pictures of the simulation‐based training. (A) Multiple professionals participated in the simulation‐based training. These include the instructor, who is an emergency certified nurse (yellow arrowhead), physicians (white arrowhead), nurses (yellow arrow), and a laboratory technologist (white arrow). (B) After each scenario, the participants receive feedback from the instructor (yellow arrowhead).

The simulation‐based training was conducted by an emergency certified nurse using a cardiopulmonary resuscitation simulation model (SaveMan Standard; KOKEN Co., Ltd., Tokyo, Japan). The training included the following three scenarios related to the knowledge test contents: “respiratory arrest/cardiac arrest,” “oversedation during sedated endoscopy,” and “anaphylaxis following pharyngeal anesthesia with lidocaine spray.” Each scenario involved 3–4 participants randomly selected from the group of physicians, nurses, and laboratory technologists (Figure 2A). The instructors verbally conveyed the changes in the simulated patient's condition, such as decreased oxygen saturation or worsening respiratory status. During each scenario, the instructors provided real‐time coaching to ensure that the participants performed high‐quality cardiopulmonary resuscitation, including appropriate chest compressions, by verifying the compression depth, rate, and full recoil displayed on the simulation model. Additional advice was given regarding the medications for cardiac arrest or anaphylaxis, automated external defibrillator (AED) use, and Code Blue activation. After each scenario, the participants received feedback from the instructor (Figure 2B).

### 2.4 Evaluated Outcomes

The changes in the correct answer rate on the knowledge tests administered before, immediately after, and at 1 month after the simulation‐based training were evaluated.

Furthermore, the knowledge test scores immediately before, immediately after, and at 1 month after training were compared among the professional groups. The knowledge test comprised the following six items: “correct chest compression,” “drugs for cardiac arrest,” “drugs for anaphylaxis,” “indications for defibrillation,” “AED location,” and “Code Blue contact number.”

The primary endpoint was the knowledge test accuracy rates immediately before, immediately after, and at 1 month after the simulation‐based training. A subgroup analysis by profession was also performed.

### 2.5 Statistical Analysis

The knowledge test accuracy rates at the three time points (before the training, immediately after the training, and at 1 month after the training) were treated as paired nominal variables and analyzed using McNemar's test. No adjustment for multiple comparisons was performed because the present study was exploratory in nature. A significance level of *p* < 0.05 was applied. All statistical analyses were performed using the Statistical Package for the Social Sciences (version 30.0; IBM Corp., Armonk, NY, USA).

## 3 Results

### 3.1 Study Participants

**FIGURE 3:**
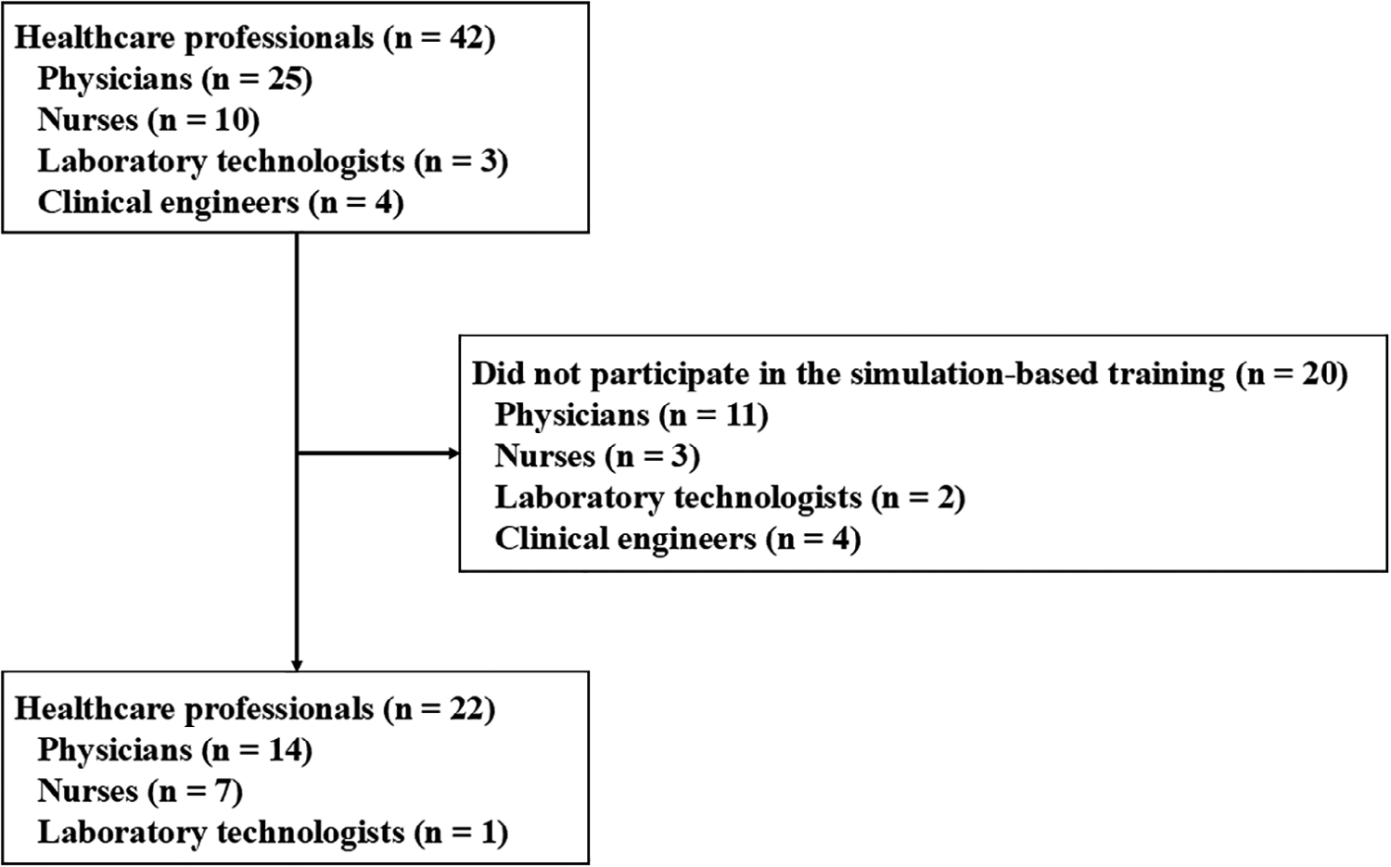
Flowchart of healthcare professionals who participated in the simulation‐based training.

During the study, approximately 42 healthcare professionals, including approximately 25 physicians, 10 nurses, three laboratory technologists, and four clinical engineers, were involved in gastrointestinal endoscopic practice at our institution (Figure 3). The details of the study participants are presented in Table 1. Altogether, 22 healthcare professionals, including 14 physicians, seven nurses, and one laboratory technologist, involved in endoscopic practice, participated in the simulation‐based training. All 22 participants who attended the simulation‐based training completed the knowledge tests before, immediately after, and at 1 month after the training. Given that only one laboratory technologist participated, this profession was excluded from the inter‐professional comparisons. Several participants had previous experience with emergency response training, including BLS, ACLS, and institution‐based emergency response training programs.

**TABLE 1:** Participant's demographics (*n* = 22).

Sex, male, *n* (%)	14 (63.6)
Profession, *n* (%)	
Physicians	14 (63.6)
Experience of physicians (years), *n* (%)	
≤ 5	3 (21.4)
6–10	3 (21.4)
11–15	4 (28.6)
≥ 16	4 (28.6)
Nurses	7 (31.9)
Clinical laboratory technologist	1 (4.5)
Experience of endoscopic practice at our institution (years), *n* (%)	
≤1	7 (31.8)
2‐5	8 (36.4)
6‐10	5 (22.7)
≧11	2 (9.1)
Time since last simulation‐based training, *n* (%)	
First time	1 (4.5)
6 months	20 (91)
1 year	1 (4.5)
Previous training courses, *n* (%)	
None	1 (4.5)
BLS	9 (41.0)
BLS and ACLS	12 (54.5)
Previous training courses by profession, *n* (%)	
Physicians	
None	1 (7.1)
BLS	1 (7.1)
BLS and ACLS	12 (85.8)
Nurses	
None	0
BLS	7 (100)
BLS and ACLS	0
Clinical laboratory technologist	
None	0
BLS	1 (100)
BLS and ACLS	0

Abbreviations: ACLS, advanced cardiovascular life support; BLS, basic life support.

### 3.2 Knowledge Test Results

**FIGURE 4:**
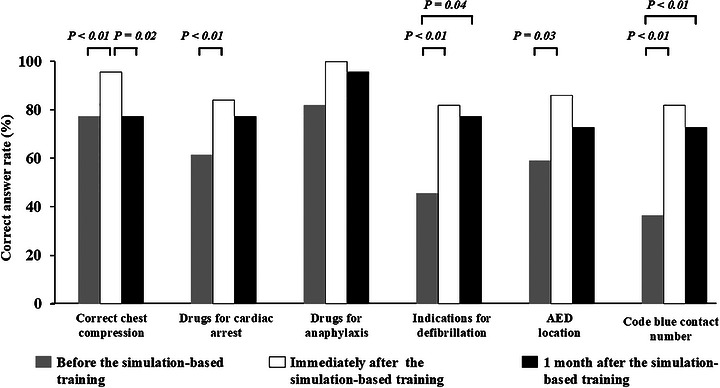
Correct answer rates for each item in the knowledge test questionnaire according to the timing of administration.

The knowledge test accuracy rates showed significant differences in the following areas: correct chest compression (before vs. immediately after, 77.3% vs.95.5%, *p* < 0.01), drugs for cardiac arrest (before vs. immediately after, 61.4% vs.84.1%, *p* < 0.01), indications for defibrillation (before vs. immediately after, 45.5% vs. 81.8%, *p* = 0.01), AED location (before vs. immediately after, 59.1% vs. 86.4%, *p* = 0.03), and Code Blue contact number (before vs. immediately after, 36.4% vs. 81.8%, *p* < 0.01) (Table S2). When comparing the results before the simulation‐based training with those obtained 1 month after the simulation‐based training, the correct response rates for the indications for defibrillation (before vs. 1 month later, 45.5% vs. 77.3%, *p* = 0.04) and the Code Blue contact number (before vs. 1 month later, 36.4% vs. 72.7%, *p* < 0.01) significantly improved. In contrast, for the other knowledge test items, no significant differences in the rates were observed between the assessments conducted before and at 1 month after the simulation‐based training (Figure 4). When comparing the knowledge test results obtained immediately after the simulation‐based training with those obtained 1 month later, the correct response rates decreased for most items. A significant decline was observed for correct chest compression (95.5% vs. 77.3%, *p* = 0.02), whereas no significant differences were observed for the other knowledge test items (Table S2).

### 3.3 Subgroup Analysis by Profession

**FIGURE 5:**
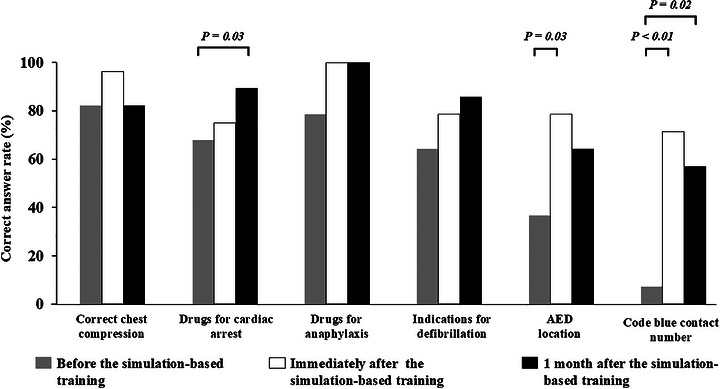
Correct answer rates for each item in the knowledge test questionnaire administered among physicians according to the timing of administration.

**FIGURE 6:**
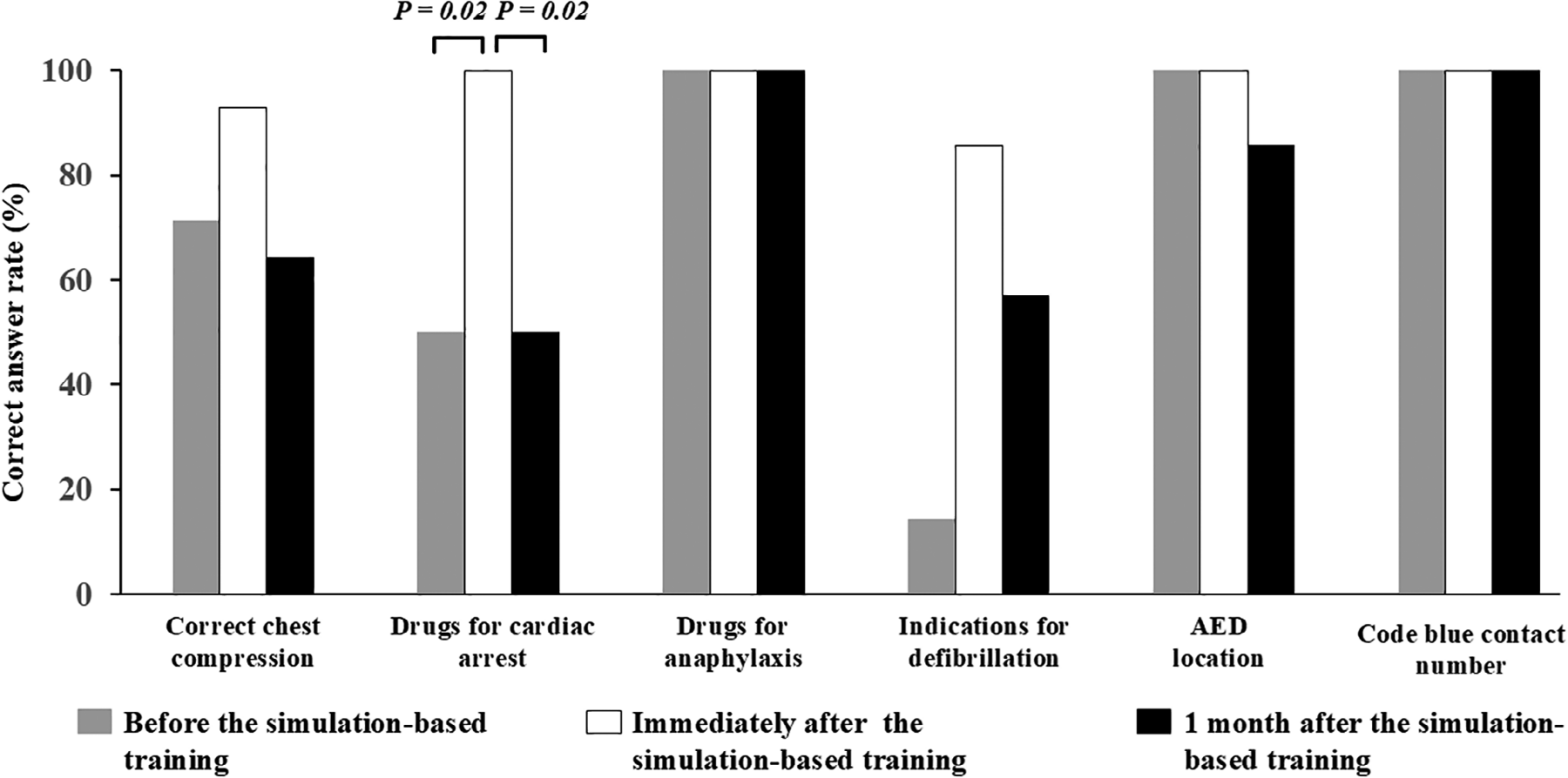
Correct answer rates for each item in the knowledge test questionnaire administered among nurses according to the timing of administration.

In the subgroup analysis involving physicians, the knowledge test accuracy rates were significantly different in the following areas: AED location (before vs. immediately after, 35.7% vs. 78.6%, *p* = 0.03), and correct Code Blue contact number (before vs. immediately after, 7.1% vs. 71.4%, *p* < 0.01) (Figure 5). In the subgroup analysis involving nurses, the rates showed significant differences in terms of the drugs for cardiac arrest (before vs. immediately after, 50% vs. 100%, *p* = 0.02) (Figure 6). Self‐reported confidence in emergency response improved after the simulation‐based training, and the proportion of participants who reported being able to use an AED increased from 81.8% before training to 90.9% immediately after training and 95.5% at 1 month after training (Table S3).

## 4 Discussions

To the best of our knowledge, the present study is the first to objectively evaluate the effectiveness of simulation‐based training for emergency response in gastrointestinal endoscopic practice using a knowledge test questionnaire administered among multiple professions, including physicians. The sedation guidelines issued by the ASGE [7], JGES, and JSA [8] recommend that, prior to sedation for gastrointestinal endoscopy, clinicians perform an American Society of Anesthesiologists Physical Status assessment, airway evaluation, and appropriate monitoring of consciousness, circulation, and respiration. To ensure preparedness for cardiac arrest scenarios, they also recommend that providers complete BLS/ACLS training. However, despite these recommendations, few studies have evaluated the effectiveness of the simulation‐based training designed for emergency response in the gastrointestinal endoscopy setting [13, 16]. The present study is unique, as it conducted scenario‐based simulation training incorporating endoscopy‐specific critical situations, including oversedation during sedated endoscopy and anaphylaxis after pharyngeal anesthesia. Furthermore, it objectively evaluated the impact of the simulation‐based training on multidisciplinary emergency response knowledge before, immediately after, and at 1 month after the training.

The study results demonstrated improvements in all knowledge test items immediately after training compared with the pre‐training assessments, except for “drugs for anaphylaxis.” However, at 1 month after the simulation‐based training, the results of most knowledge test items did not differ significantly from the pre‐training assessment rates, except for “indications for defibrillation” and “Code Blue contact number”. Previous studies conducted in other clinical fields have similarly reported declines in emergency response knowledge levels within 1–6 months after the training [19, 20, 21]. Our findings suggest that the educational effect of the simulation‐based training in gastrointestinal endoscopy diminishes within approximately 1 month, consistent with the findings in other disciplines. Regular participation in simulation‐based training reportedly promotes sustained knowledge retention [19, 20, 22], and our study results support the need for repeated training to maintain educational benefits in gastrointestinal endoscopy.

In the subgroup analysis involving physicians, the items assessing BLS knowledge, such as correct chest compression, drugs for cardiac arrest, drugs for anaphylaxis, and indications for defibrillation, already showed high accuracy rates before the simulation‐based training, and no significant improvements in the results were observed immediately after the training. Contrarily, significant improvements were observed in the knowledge items related to the local clinical environment, namely AED location and Code Blue contact number. Conversely, among nurses, the accuracy rates for AED location and Code Blue contact number were already 100% before the simulation‐based training, whereas for the drugs for cardiac arrest, significant improvement was observed immediately after the training compared with the pre‐training assessment results. These findings may reflect the professional characteristics of physicians and nurses. Physicians reportedly have higher baseline knowledge of BLS than nurses [23], which is consistent with the higher level of BLS knowledge observed among physicians in the present study. The higher accuracy rates among nurses for “AED location” and “Code Blue contact number” in the present study may reflect the nature of their routine duties, which often involve working in recovery areas where AEDs are located and initiating Code Blue activation during emergencies. Multidisciplinary simulation‐based training that exposes participants to shared scenarios may help reduce disparities in knowledge across professions. Such harmonization of knowledge and improved team‐wide information sharing are particularly important to ensure coordinated responses during emergencies. In endoscopy units, where team composition frequently changes, multidisciplinary simulation‐based training is considered essential for establishing systems that function independently of specific personnel or roles.

The present study has several limitations. First, the present investigation was a single‐center observational study with a small sample size, which may limit the generalizability of the findings, increase the risk of selection bias, and reduce the statistical robustness of the analyses, potentially increasing the likelihood of false‐positive findings. Second, the post‐training evaluation period was relatively short (1 month), thereby limiting our ability to assess the long‐term educational effects. Third, the same knowledge test was repeatedly administered at all three time points, which may have introduced a testing effect. Fourth, the knowledge test was developed based on institutional emergency response protocols and was not externally validated; moreover, knowledge‐based assessment alone may not fully reflect actual behavioral performance during emergencies or patient outcomes. Therefore, further multicenter studies with larger sample sizes and longer follow‐up periods are warranted.

In conclusion, our simulation‐based training for emergency response in gastrointestinal endoscopy improves knowledge in the short term. However, this effect diminished within 1 month after the training, indicating the need for repeated simulation‐based training to sustain its long‐term educational effects. Additionally, multidisciplinary simulation‐based training improved the knowledge in areas that were relatively weak for each profession, resulting in comparable knowledge levels between physicians and nurses. Thus, our multidisciplinary simulation‐based training program reduced the inter‐professional disparities and may be useful for standardizing emergency response knowledge across roles. Future studies should include long‐term follow‐up investigation and examine associations with real‐world clinical outcomes.

## Author Contributions

*Conceptualization*: Mitsuru Otsuka and Tsunetaka Kato. *Methodology*: Mitsuru Otsuka, Tsunetaka Kato, Takuto Hikichi, and Masao Kobayakawa. *Formal analysis*: Mitsuru Otsuka, Tsunetaka Kato, and Takuto Hikichi. *Investigation*: Mitsuru Otsuka, Tsunetaka Kato, Takuto Hikichi, Jun Nakamura, Takumi Yanagita, Eisuke Kameoka, Daiki Nemoto, Rei Suzuki, Mitsuru Sugimoto, Hiroyuki Asama, Hiroshi Shimizu, Kento Osawa, and Rei Ohira. *Data curation*: Mitsuru Otsuka, Tsunetaka Kato, and Takuto Hikichi. *Writing – original draft preparation*: Mitsuru Otsuka, Tsunetaka Kato, and Takuto Hikichi. *Writing – review and editing*: Jun Nakamura, Takumi Yanagita, Eisuke Kameoka, Daiki Nemoto, Rei Suzuki, Mitsuru Sugimoto, Hiroyuki Asama, Hiroshi Shimizu, Kento Osawa, Rei Ohira, Masao Kobayakawa, and Hiromasa Ohira. *Supervision*: Hiromasa Ohira. *Project administration*: Takuto Hikichi and Tsunetaka Kato. All authors have read and approved the final version of the manuscript.

## Funding

The authors have nothing to report.

## Ethics Statement

**Approval of the research protocol by an Institutional Review Board**: N/A

## Consent

Obtained from all participants.

## Conflicts of Interest

The authors declare no conflicts of interest.

## Supporting information




**Supporting file 1**: deo270372‐sup‐0001‐SuppMat.docx
