# Supplementary material for: Effectiveness of Simulation‐based Training on Emergency Response Knowledge Among Inter‐Professional Staff Involved in Gastrointestinal Endoscopic Practice
Source: DEN Open. 2026 Jul 1;7(1):e70372. doi: 10.1002/deo2.70372 (PMC13322650; doi:10.1002/deo2.70372)
Supplement: Supplementary file 1 — Supporting file 1: deo270372‐sup‐0001‐SuppMat.docx [file DEO2-7-e70372-s001.docx]

**Supplementary Table S1.** **Questionnaire and knowledge test on simulation-based training on emergency response**

| Questionnaire on simulation-based training on emergency response: Q1-Q4 |
| --- |
| Q1. Please check any training courses you have previously attended. (multiple answers allowed) |
| AED |
| BLS |
| ACLS |
| None |
| Q2. When did you last participate in a simulation-based training session? |
| First time |
| 6 months ago |
| 1 year ago |
| 2 years ago |
| More than 2 years ago |
| Q3. Are you confident in responding to an emergency situation? |
| Very confident |
| Somewhat confident |
| Somewhat not confident |
| Not confident |
| Q4. Are you able to use an AED? |
| Yes |
| No |
| Knowledge test on simulation-based training on emergency response: Q5-Q10 |
| Q5. Please select the appropriate chest compression rate for adults. |
| 60–80 compressions/min |
| 80–100 compressions/min |
| 100–120 compressions/min |
| 130–150 compressions/min |
| Q6. Please select the appropriate chest compression depth for adults. |
| 2 cm |
| 3 cm |
| 5 cm |
| 8 cm |
| Q7. Where is the AED located in the endoscopic department and fluoroscopy room (descriptive questions)? |
| Endoscopic department: ( ) |
| Fluoroscopy room: ( ) |
| Q8. Please check the ECG rhythms that require electrical defibrillation (multiple answers allowed). |
| Pulseless VT |
| Asystole |
| PEA |
| VF |
| Atrioventricular block |
| None |
| Q9. For each of the following situations, please list the medication to be used from the emergency carts in the endoscopic department and fluoroscopy rooms. (descriptive questions) |
| One primary drug prepared for cardiac arrest |
| One primary drug prepared for VF/VT |
| One drug administered for asystole/PEA |
| One drug administered first for anaphylaxis |
| Q10. What is the telephone number to activate a Code Blue? (descriptive questions) |
| ( ) |

AED, automated external defibrillator; BLS, basic life support; ACLS, advanced cardiovascular life support; ECG, electrocardiogram; VT, ventricular tachycardia; PEA, pulseless electrical activity; VF, ventricular fibrillation

**Supplementary Table S2. Overall and professional-specific knowledge test correct answer rates**

|  | Correct answer rate (%) | | | *P*-value | | |
| --- | --- | --- | --- | --- | --- | --- |
| Knowledge test items | Pre-simulation-based training | Post-simulation-based training | 1 month after simulation-based training | Pre-simulation-based training vs. post-simulation-based training | Post-simulation-based training vs. 1 month after simulation-based training | Pre-simulation-based training vs. 1 month  after |
| Correct chest compression | 77.3 | 95.5 | 77.3 | < 0.01 | 0.02 | 1.00 |
| Physicians (n=14) | 82.1 | 96.4 | 82.1 | 0.13 | 0.13 | 1.00 |
| Nurses (n=7) | 71.4 | 92.9 | 64.3 | 0.25 | 0.22 | 1.00 |
| Drugs for cardiac arrest | 61.4 | 84.1 | 77.3 | < 0.01 | 0.55 | 0.09 |
| Physicians (n=14) | 67.9 | 75.0 | 89.3 | 0.50 | 0.13 | 0.03 |
| Nurses (n=7) | 50,0 | 100 | 50.0 | 0.02 | 0.02 | 1.00 |
| Drugs for anaphylaxis | 81.8 | 100 | 95.5 | 0.13 | 1.00 | 0.25 |
| Physicians (n=14) | 78.6 | 100 | 100 | 0.25 | 1.00 | 0.25 |
| Nurses (n=7) | 100 | 100 | 100 | 1.00 | 1.00 | 1.00 |
| Indications for defibrillation | 45.5 | 81.8 | 77.3 | < 0.01 | 1.00 | 0.04 |
| Physicians (n=14) | 64.3 | 78.6 | 85.7 | 0.50 | 1.00 | 0.25 |
| Nurses (n=7) | 14.3 | 85.7 | 57.1 | 0.06 | 0.63 | 0.38 |
| AED location | 59.1 | 86.4 | 72.7 | 0.03 | 0.25 | 0.38 |
| Physicians (n=14) | 35.7 | 78.6 | 64.3 | 0.03 | 0.5 | 0.13 |
| Nurses (n=7) | 100 | 100 | 85.7 | 1.00 | 1.00 | 1.00 |
| Code blue contact number | 36.4 | 81.8 | 72.7 | < 0.01 | 0.63 | <0.01 |
| Physicians (n=14) | 7.1 | 71.4 | 57.1 | < 0.01 | 0.63 | 0.02 |
| Nurses (n=7) | 100 | 100 | 100 | 1.00 | 1.00 | 1.00 |

AED, automated external defibrillator

**Supplementary Table S3. Self-reported confidence in emergency response and ability to use an automated external defibrillator**

|  | Pre-simulation-based  training (%) | Post-simulation-based  training (%) | 1 month after simulation-based training (%) |
| --- | --- | --- | --- |
| Confidence in emergency response |  |  |  |
| Very confident | 0 | 9.1 | 0 |
| Somewhat confident | 18.2 | 40.9 | 45.5 |
| Somewhat not confident | 36.4 | 36.4 | 40.9 |
| Not confident | 45.4 | 13.6 | 13.6 |
| Ability to use an AED (yes) | 81.8 | 90.9 | 95.5 |

AED, automated external defibrillator
